# Lymphocyte Transformation Test Based on Lymphocyte Changes Observed by a Hematology Analyzer before and after Phytohemagglutinin Stimulation

**DOI:** 10.1155/2022/5967429

**Published:** 2022-11-07

**Authors:** Lulu Zhang, Tinghua Feng, Hui Liu

**Affiliations:** College of Medical Laboratory, Dalian Medical University, Dalian, China

## Abstract

**Objective:**

The lymphocyte transformation test is a classical test for the detection of cellular immune function and is based on subjective judgment. In this study, we have established an objective novel lymphocyte transformation test using the hematology analyzer to observe lymphocyte transformation.

**Methods:**

Whole blood cells were cultured using a whole blood method with a lymphocyte culture medium; phytohemagglutinin was used to stimulate the experimental samples, and control was set up at the same time. After the whole blood cells were cultured, the number of lymphocytes in the two groups was observed using a hematology analyzer, and the conversion rate was calculated. The new method was used to observe differences in lymphocyte conversion in the peripheral blood of patients with hematopathy and healthy persons.

**Results:**

There were significant differences between the stimulated peripheral blood group and the blank group. The transformation rate of peripheral blood lymphocytes in patients with hematopathy was significantly lower than that in healthy persons; the difference was statistically significant (*P* < 0.05).

**Conclusion:**

Lymphocyte transformation can be observed using a hematology analyzer. The lymphocyte transformation test that is based on the determination of lymphocyte count by a hematology analyzer has important clinical value.

## 1. Introduction

There are two types of immune response in humans [1]. One arm is the humoral immune response, involving B cells that recognize circulating antigens or pathogens in the lymph or blood, and the second arm is the cell-mediated immune response that mainly involves T cells and responds to viral cells, tumor cells, or transplants [2].

Cellular immunity is an important type of adaptive immune response [3]. The biological significance of this type of immunity is that immune cells can effectively eliminate antigenic foreign bodies in the body by recognizing “self” and “non-self” to maintain the relative stability of the internal environment of the body [4]. The evaluation of cellular immune function mainly includes assessing the lymphocyte count and lymphocyte function test [5]. Counting the number of lymphocytes includes the detection of CD antigen and classification and counting of lymphocyte subsets. Such methods only reflect the number of lymphocytes, but not their function; the detection of which is more important.

In vitro measurement of cellular immune function requires firstly obtaining lymphocytes from peripheral blood of humans or animals, followed by subsequent experiments, including the T lymphocyte proliferation test. The lymphocyte transformation test is a method to determine the function and state of immune cells in a certain organism by stimulating the proliferation of lymphocytes [6]. The principle of this test is that the metabolism and morphology of T cells change after being stimulated by antigens or mitogens in vitro, which is mainly manifested as increases in protein and nucleic acid synthesis, a series of proliferation reactions, and transformation into lymphoblasts [7]. The T lymphocyte transformation rate reflects the cellular immune status of the body [8].

The lymphocyte transformation test technique mainly involves morphological examination, the ^3^H-TdR method and methyl thiazolyl tetrazolium colorimetric analysis (MTT assay) [9, 10]. The ^3^H-TdR approach works on the principle that DNA synthesis involves ^3^H-TdR absorption during transformation of T cells into lymphoblastic cells, and the degree of transformation is proportional to uptake of ^3^H-TdR [11]. Although the ^3^H-TdR is objective and accurate, there is a potential danger of radioactive contamination; it also requires certain equipment conditions. The MTT has a strong nonspecific reaction and high background [12]. The results of the morphological examination are greatly influenced by subjective factors, and its reproducibility is poor; hence, low efficiency of determination is inevitable. Therefore, none of the mentioned methods is ideal, which impedes the evaluation of cellular immune function. In this study, we attempt to conduct lymphocyte transformation tests based on morphology using a hematology analyzer so as to make the findings more objective, trustworthy, and stable.

## 2. Methods

The study was conducted in accordance with the principles and guidelines laid down in the Declaration of Helsinki. The Dalian Medical University Ethics Committee approved the study and waived the requirements for written informed consent, since the samples were remnants following clinical use and therefore not specifically collected for this study and no risk to patients was involved.

**Figure 1:**
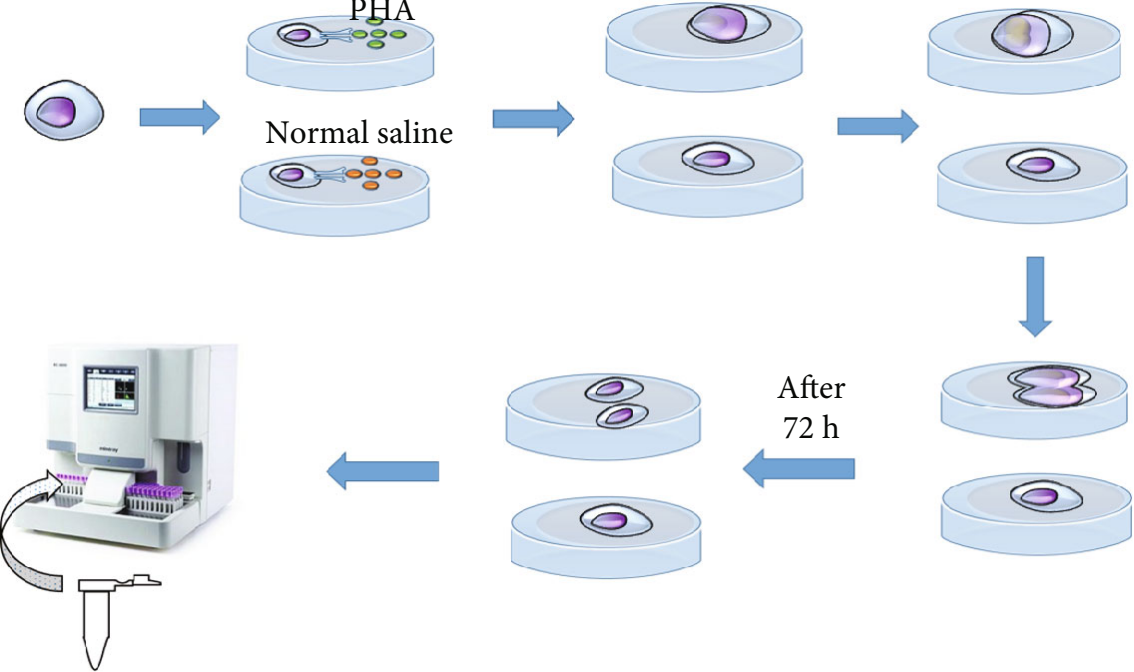
The lymphocyte transformation using a hematology analyzer.

Lymphocyte function was assessed by measuring lymphocyte proliferation in response to mitogen phytohemagglutinin (PHA) [13]. T cells were stimulated in vitro using a T lymphocyte-sensitive stimulant, such as phytohemagglutinin-P (PHA-P) [14, 15]. T cells undergo morphological and biochemical alterations when activated. Some small lymphocytes are transformed into immature lymphocytes or blast cells, which undergo mitosis and proliferation. The percentage of lymphocytes was found to increase when the automated hematology analyzer calculated the proportion of lymphocytes. As a result, the rate of lymphocyte transformation can be measured (Figure 1).

### 2.1. Morphological Method

Using the whole blood method, 1.8 mL of 10% bovine serum was added to the RPMI 1640 culture medium, and then, 0.2 mL of EDTA anticoagulated whole blood (sample to be tested) along with 0.1 mL PHA-P (Solarbio origin: Beijing article number: p8090 specification: 5 mg) (1 mg/mL) was added to the culture dish. After 72 hours of culture in a 5% CO_2_ incubator, some of the cells were centrifuged and the supernatant was removed. A smear was made and stained with Giemsa stain, the morphological changes in lymphocytes were observed under a microscope, and the percentage of lymphoblasts in 200 lymphocytes was calculated.

### 2.2. Hematology Analyzer Method

We added 160 *μ*L of 1640 culture medium (10% bovine serum), 40 *μ*L of EDTA anticoagulated whole blood (sample to be tested), and 20 *μ*L of PHA-P (1 mg/mL) into a 96-well clear flat bottom TC-treated microculture plate; each sample (220 *μ*L) was tested three times in three wells of the microculture plate at the same time (Table 1). At the same time, in the blank or control sample, PHA was replaced with normal saline. Subsequently, culture in a 5% CO_2_ incubator for 72 hours was performed, mixed, and blood cells were tested with an automated hematology analyzer (Mindray, Shenzhen, China).

The lymphocyte transformation rate is calculated as follows:

(1) $$\begin{array}{c} \frac{\text{lym}\%\left(\text{PHA group}\right)-\text{lym}\%\left(\text{blank group}\right)}{\text{lym}\%\left(\text{PHA group}\right)}\times 100\%. \end{array}$$

### 2.3. Hematology Analyzer and Morphological Comparison

A total of 55 healthy individuals were randomly selected from the Lushun People's Hospital in Dalian, China. Individuals in this random sample were aged between 20 and 40 years (27 males and 28 females). For the lymphocyte transformation test, whole blood collected in EDTA-coated tubes was used to obtain a complete blood count using a hematology analyzer. All experiments were carried out using the same procedure (Table 1). The results were compared to those produced using the morphological counting approach and analyzed.

### 2.4. Stability Test

A healthy volunteer was chosen from whom peripheral blood was collected for five consecutive days in EDTA-coated tubes, and complete blood counts were determined using a hematology analyzer. All experiments were carried out using the same protocol (Table 1). The volunteer followed the same routine so as to maintain the same physiological condition for five consecutive days. To assess the stability of the hematology analyzer measurements, the coefficient of variation (CV) was computed.

### 2.5. Gender Comparison Testing

A random sample of 22 healthy men and 25 healthy women was obtained from the Lushun People's Hospital in Dalian, China. Individuals in this study's random sample were aged between 20 and 40 years. Venous blood samples were collected in EDTA-anticoagulated tubes. The experimental procedure was the same as described in Table 1. The results of lymphocyte transformation in males and females were compared.

### 2.6. Age Group Comparison

The study involved 20 healthy subjects, aged >60 years (mean age, 84 years), 30 healthy subjects, aged 20 through 40 years, with equal distribution of men and women. The random samples were provided by the Lushun People's Hospital in Dalian, China. The experimental procedure was the same as described in Table 1. The results of lymphocyte transformation in different age groups were compared.

### 2.7. Clinical Specimen Test

The hematological disease group, which included leukemia and malignant lymphoma, was classified according to the World Health Organization classification of hematological malignancies. The inclusion criteria for leukemia were as follows: hospitalized patients with ≥20% blasts in blood or bone marrow smear and age > 18 years [16]. A total of 28 participants (20 males and 8 females; age range, 29–74 years; mean age, 51 years) in the leukemia group were included. The inclusion criteria for malignant lymphoma were as follows: hospitalized patients who on lymph node biopsy were diagnosed lymphoma (Hodgkin's or non-Hodgkin's) and age>18 years [16]. A total of 16 participants (9 males and 7 females; age range, 25–77 years; mean age, 60 years) in the malignant lymphoma group were included.

The normal blood samples were obtained from healthy individuals subjected to routine physical exams. The inclusion criteria were routine blood tests and blood biochemical tests were within the normal range and age > 18 years. A total of 30 participants (14 males and 16 females; age range, 25–84 years; mean age, 60 years) in the healthy group were included. The samples were collected from the second department of the First Affiliated Hospital of Dalian Medical University. The experimental procedure was the same as mentioned.

### 2.8. Statistical Analysis

Analysis of variance was used to compare the means of multiple groups; an independent sample *t*-test was used to compare the mean of the single group; K-S analysis and Q-Q chart were used to test the normal distribution. Statistical significance was set at *P* < 0.05. SPSS 17.0 (IBM, Armonk, New York, USA) statistical software was used for the mentioned analysis.

## 3. Results

### 3.1. Morphological Count and Hematology Analyzer

**Figure 2:**
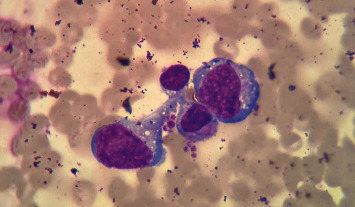
Whole blood was stimulated using PHA-P and cultured for 72 h (morphological counting method).

Lymphocyte morphological alterations were observed by morphological counting under a microscope (Figure 2). The lymphocyte transformation rate was found to be 60%–80% after counting.

The lymphocyte ratios were detected using the hematology analyzer (Table 2). The lymphocyte ratios of 55 healthy persons in the experimental group were significantly higher than those in the control group (*P* < 0.001). Table 2 presents the lymphocyte conversion rates of 55 healthy individuals as measured by the hematology analyzer. The results obtained by the hematology analyzer method were essentially similar to the lymphocyte transformation rate as determined by the morphological counting method.

### 3.2. Stability Results

For the same volunteer, the lymphocyte transformation rate in the peripheral blood was measured for five consecutive days (Table 3). The lymphocyte transformation rate was found to be between 60% and 80% and the coefficient of variation (CV) was 6%.

### 3.3. Lymphocyte Transformation Tests according to Gender

In the gender comparison experiment, the mean percentages of lymphocytes in the male blank and experimental groups were 14.848 and 37.276, respectively. The mean percentages of lymphocytes in the female blank and experimental groups were 20.409 and 34.605, respectively. The lymphocyte conversion rates of males and females are shown in Table 4. The transformation rates of the two groups were similar, with no significant differences (*P* = 0.385).

### 3.4. Lymphocyte Transformation Tests according to Age

In the comparison experiment for different age groups, the average percentage of lymphocytes in the blank group of the younger age group was 15.300 and in the experimental group was 36.973. The average percentage of lymphocytes in the blank group of the elderly group was 17.840 and in the experimental group was 35.455. The lymphocyte conversion rates of the younger and elderly groups are shown in Table 5. The conversion rates of the two groups were similar with no significant differences (*P* = 0.413).

### 3.5. Normal Distribution Test of Blood Samples in Patients and Healthy Persons

**Figure 3:**
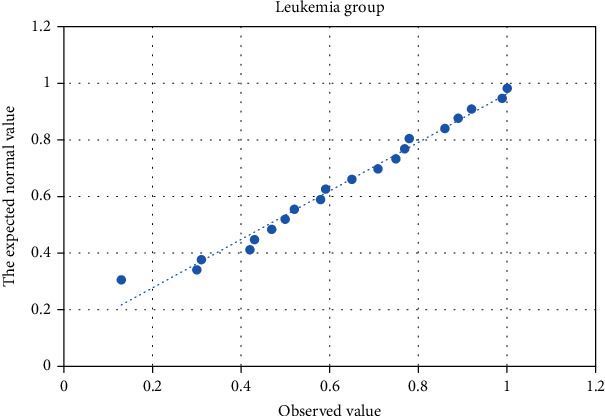
Normal distribution test Q-Q chart of the leukemia group.

**Figure 4:**
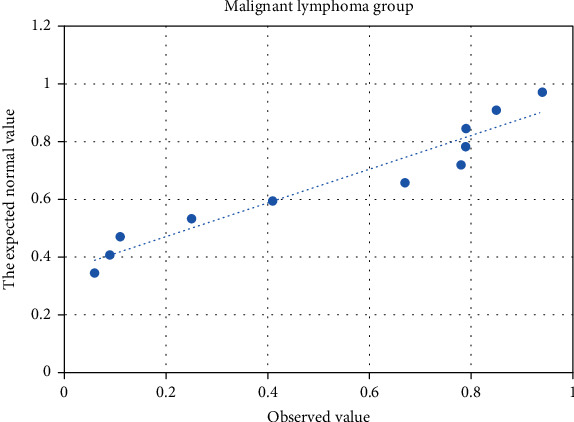
Normal distribution test Q-Q chart of the malignant lymphoma group.

**Figure 5:**
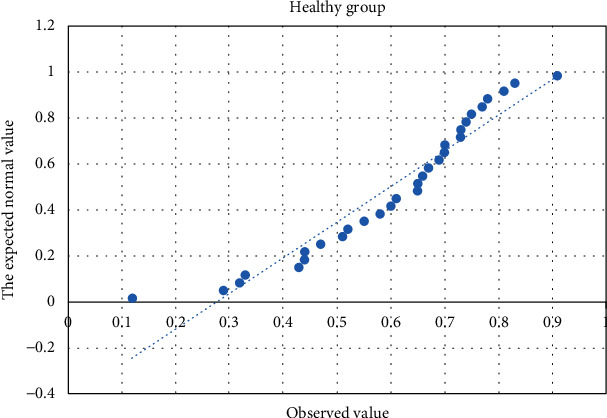
Normal distribution test Q-Q chart of the healthy group.

Forty-four patients' (28, leukemia; 16, malignant lymphoma) and 30 healthy persons' data were analyzed by normal distribution Q-Q chart (Figures 3–5). *P* values of the normal distribution K-S test in the leukemia, lymphoma, and healthy groups were 0.305, 0.272, and 0.641, respectively; *P* values were greater than 0.05, indicating that the three groups were all in normal distribution.

### 3.6. Differences between Hematopathy Patients and Healthy Persons

We compared the hematopathy group with the healthy group (Table 6). There was a significant difference in the mean lymphocyte transformation rate between patients with hematopathy and healthy people (^∗∗^*P* < 0.01).

### 3.7. Patients with Different Hematopathies and Healthy Persons

We compared the leukemia group and the lymphoma group with the healthy group (Table 7). The differences between the average lymphocyte transformation rate of the leukemia and healthy groups (^∗^*P* < 0.05) and those between the lymphoma group and the healthy group (^∗∗^*P* < 0.01) were statistically significant.

## 4. Discussion

The hematological analyzer is one of the most commonly used instruments for the detection and analysis of blood abnormalities in medical laboratories at present [17, 18]. It is easy to operate, fast to detect blood anomalies, and reports many parameters. The results are accurate and reliable, which greatly improves the efficiency and quality of hematological analysis [19]. In this experiment, the lymphocyte transformation test was based on the analysis of blood cells. The results were more accurate. Because morphology is greatly influenced by subjective factors, poor repeatability, and low detection efficiency when observing and counting lymphoblasts, the correlation between the morphological method and the hematology analyzer was not performed. However, all transformed lymphocytes observed under the microscope could be measured by the hematology analyzer. The results obtained by the automated hematology analyzer are largely similar to the morphological method, thereby implying that the automated hematology analyzer can accurately reflect lymphocyte transformation rate. The results of the stability test obtained by the automated hematology analyzer revealed CV < 10%, indicating that the test is repeatable.

We studied the lymphocyte transformation test data from many healthy men and women to assess if the experiment is modified by sex and age. Lymphocyte transformation rates in males and females were comparable, with no significant variation (*P* > 0.05). Next, we examined the lymphocyte transformation test findings of people aged between 20 and 60 years. The young and old groups' lymphocyte transformation rates were similar, with no significant differences (*P* > 0.05). These results show that gender and age do not affect lymphocyte transformation rates.

Currently, phytohemagglutinin (PHA) and concanavalin A (ConA) are some of the most often employed mitotic stimulators in lymphocyte transformation experiments [20, 21]. PHA outperformed all other mitogens in inducing mitosis in human peripheral blood cultures in experiments where each mitogen was tested individually. Furthermore, in the combined use of two mitogens, there was no reliable combination to enhance mitotic stimulation [22]. PHA, a mitotic lectin that binds to the TCR on T cell surfaces, is identical to the receptor that recognizes APC surface ligand [23]. The signaling cascade it initiates in lymphocytes can activate particular molecules, such as p38-MAPK or the transcription factor family and signal transducer and activator of transcription (STAT) pathway [24], resulting in cell cycle entry and DNA replication [25].

This experiment provides good evidence of T cell abnormality. Traditionally, RPMI 1640 culture medium (10% calf serum should be prepared before use) and PHA-P stimulant (1000 *μ*g/mL was prepared with RPMI 1640 culture medium) were commonly used for lymphocyte transformation test, which involves culturing the test sample under the CO_2_ cell culture shelves for 72 hours and observing and counting the results under a microscope. We observed that the hematology analyzer could check lymphocyte transformation through the change in lymphocyte count, to make the lymphocyte transformation experiment more stable and objective, evaluate the cellular immune function of the body more accurately, and provide reliable indicators for disease diagnosis and related research.

Hematological diseases originate primarily in the hematopoietic system [26]. There are various kinds of blood disorders that can be broadly divided into three categories: red cell diseases, white cell diseases, and hemorrhagic and thrombotic disorders. Common hematological diseases include leukemia [27], multiple myeloma, and lymphoma [28]; in blood diseases, changes in lymphocytes and immune function are seen. Malignant cloning of hematopoietic stem cells in patients with leukemia will lead to an increase in the number of primitive blood cells and thereby decreased cellular immune function [29, 30]. In malignant lymphomas, large numbers of proliferating abnormal lymphocytes lack proliferative activity after antigen and mitogen stimulation, which will lead to decreased cellular immunity [31, 32]. Therefore, the lymphocytes from leukemia and malignant lymphoma patients may have a lower transformation rate compared to healthy people. The lymphocyte transformation test is a routine test to detect cellular immune function. If the lymphocyte transformation rate is taken as the evaluation standard, the lymphocyte transformation rate of patients with hematopathy would be lower than that of healthy persons on analysis with a hematology analyzer, which indicates that there is a significant difference in the lymphocyte transformation rate between the patients with hematopathy and healthy persons; the cellular immune function of patients with hematopathy is obviously decreased. This implies that the method of detecting lymphocyte transformation by a hematology analyzer is correct; this has clinical significance for the observation, diagnosis, and treatment of blood diseases.

Although this study has numerous strengths, it also has some limitations. First, tritium is strictly controlled in China because it is highly radioactive. Therefore, the ^3^H-TdR incorporation method could not be carried out in this experiment, and the experimental results were not compared with the ^3^H-TdR incorporation method. Second, because the experimental samples could not be preserved for a long time, we could not continuously measure the lot-to-lot variability of the samples. However, we measured the same volunteer for five consecutive days, and the coefficient of variation of the measured value for five consecutive days was 6%, which shows that our experiment has a relatively good stability.

In conclusion, it is feasible to use a hematology analyzer for the lymphocyte transformation test. The observation is more accurate and convenient, and the experimental data are more accurate as compared with conventional methods. This novel method can be used in the diagnosis and treatment of clinical diseases and related research. Therefore, the determination of lymphocyte transformation by a hematology analyzer has important clinical value.

## Figures and Tables

**Table 1:** The trial dose added to each hole in the lymphocyte transformation test.

Group	10% 1640 (*μ*L)	Blood (*μ*L)	PHA-P (*μ*L)	Saline (*μ*L)
Blank	160	40	0	20
Test	160	40	20	0

**Table 2:** The result of lymphocyte transformation in a healthy person.

Group	*N*	Lympercent (%)			Lymphocyte transformation rate (%)
		Mean	SD	*P*	
Blank	55	15.65	9.46	<0.001	57
PHA	55	37.04	12.65		

**Table 3:** Test results for five consecutive days.

Date	Rate (%)	Mean (%)	SD	CV (%)
First	77	74	4.8	6
Second	80			
Third	70			
Fourth	69			
Fifth	72			

**Table 4:** Analysis of results of lymphocyte transformation test in male and female.

Group	*N*	Transformation rate (%)	SD	*t*	*P*
Males Females	22 25	48 56	0.32 0.28	0.878	0.385

**Table 5:** Analysis of results of lymphocyte transformation tests in various age groups.

Group	*N*	Transformation rate (%)	SD	*t*	*P*
Young adults Aged adults	30 20	56 48	0.28 0.36	0.825	0.413

**Table 6:** Transformation rate of hematopathy patients and healthy persons ($\bar{X}\pm S$).

Group	*N*	Transformation rate (%)	SD	*t*	*P*
Hematopathy Healthy	44 30	42 60	0.36 0.18	2.870	0.005

**Table 7:** Transformation rate of different hematopathy patients and healthy persons ($\bar{X}\pm S$).

Group	*N*	Transformation rate (%)	SD	*t*	*P*
Leukemia group	28	45	0.35	2.048	0.045
Lymphoma group	16	36	0.38	2.936	0.005
Healthy group	30	60	0.18	—	—

## Data Availability

The datasets generated and/or analyzed during the current study are not publicly available but are available from the corresponding author on reasonable request.
